# Association of the tumour stroma percentage in the preoperative biopsies with lymph node metastasis in colorectal cancer

**DOI:** 10.1038/s41416-019-0671-7

**Published:** 2019-12-02

**Authors:** Meiting Fu, Dexin Chen, Fuzheng Luo, Mengshu Li, Yadong Wang, Junsheng Chen, Aimin Li, Side Liu

**Affiliations:** 1 Guangdong Provincial Key Laboratory of Gastroenterology, Department of Gastroenterology, Nanfang Hospital, Southern Medical University, Guangzhou, Guangdong 510515 China; 2 Department of General Surgery, Nanfang Hospital, Southern Medical University, Guangzhou, Guangdong 510515 China

**Keywords:** Predictive markers, Cancer microenvironment, Colorectal cancer

## Abstract

**Background:**

Preoperative prediction of lymph node (LN) status is integral to determining the most appropriate treatment strategy for colorectal cancer (CRC). This study aimed to develop and validate a nomogram to predict LN metastasis in CRC preoperatively.

**Methods:**

A total of 530 patients were enrolled and divided into training and validation cohorts. The tumour stroma percentage (TSP) of the preoperative biopsies was assessed. The risk factors for LN metastasis were selected, and a nomogram was constructed subsequently. The performance of the nomogram was assessed by using the AUROC and the calibration curve, and then validated in the validation cohort.

**Results:**

High TSP was significantly associated with LN metastasis in both the training and validation cohorts. Computed tomography (CT)-reported T stage, CT-reported LN status, preoperative tumour differentiation, carcinoembryonic antigen, carbohydrate antigen 19-9 and TSP were independent predictors of LN metastasis in CRC. A nomogram incorporating the six predictors was constructed. The nomogram yielded good discrimination and calibration, with an AUROC of 0.846 (95% CI: 0.807−0.886) and 0.809 (95% CI: 0.745−0.872) in the training and validation cohorts, respectively.

**Conclusions:**

Assessment of TSP in the preoperative biopsies provided additional information about the LN status. The nomogram was useful for tailored therapy in CRC preoperatively.

## Introduction

Colorectal cancer (CRC) is the third most commonly occurring cancer and the second leading cause of cancer-related death globally.^1^ Preoperative prediction of lymph node (LN) metastasis is important for clinical practice, as it provides valuable information about the prognosis and treatment strategy decisions for CRC, including the administration of neoadjuvant and/or adjuvant therapy and the adequacy of surgical resection.^2,3^ Several histopathological parameters, such as lymphovascular infiltration and depth of tumour invasion, were reported as the predictors of LN metastasis;^4^ however, these parameters are only available after surgery. So far, imaging modalities, including computed tomography (CT), are commonly used for evaluating the LN status in the clinic. Unfortunately, their overall accuracy in determining the LN status is oftentimes limited.^5–7^ Therefore, a robust biomarker is needed to improve the predictive performance of current strategies for LN metastasis preoperatively in patients with CRC.

The tumour microenvironment comprises the extracellular matrix surrounding the tumour cells and other nonneoplastic cells, such as fibroblasts and immune cells. It plays a critical role in tumour cells behaviour and disease progression.^8,9^ Recently, tumour stroma has been identified as an important determinant of metastasis of tumour cells.^10^ An increased proportion of tumour stroma was associated with unfavourable oncological outcomes in several solid tumours.^11–13^ The tumour stroma percentage (TSP) could be assessed easily by using the haematoxylin and eosin (H&E)-stained sections of specimens, and was proved to be a strong and independent prognostic parameter.

In clinical practice, CRC is routinely diagnosed using the preoperative biopsies obtained from colonoscopy and then processed using H&E staining. These biopsies are adequate to confirm the suspected malignancy before surgery. It would be helpful to have a diagnostic biomarker to predict LN status from the biopsies. Previous studies revealed that the TSP of the preoperative biopsies helped in predicting the metastasis of prostate and oesophageal cancer.^14,15^ However, the role of TSP in the preoperative biopsies of CRC has not yet been investigated.

Therefore, this study aimed to investigate the predictive value of TSP in the preoperative biopsies for the LN status in CRC. Furthermore, a non-invasive nomogram incorporating TSP and potential clinicopathological risk factors was developed and validated to individually estimate the likelihood of LN metastasis preoperatively.

## Methods

### Study population

The training cohort was retrospectively collected from the institutional database for medical records between January 1, 2016 and June 30, 2018 for developing the prediction model. The inclusion criteria were as follows: pathologically confirmed stage I–III CRC underwent surgical resection with curative intent, lymphadenectomy performed with at least 12 LNs harvested and complete clinicopathological data. Patients with double or multiple primary tumours and who received neoadjuvant therapy (including chemotherapy, radiotherapy and chemoradiotherapy) were excluded. Finally, 353 consecutive patients were included (Supplementary Fig. S1). From October 1, 2014 to December 31, 2015, an independent validation cohort of 177 consecutive patients was included using the same criteria as those for the training cohort to validate the predictive performance of the model. Two cohorts were disconnected based on date.

The baseline clinicopathological information, including patients’ demographics (age and sex), tumour location, pathological characteristics of biopsies (tumour differentiation and histological type), tumour markers [carcinoembryonic antigen (CEA) and cancer antigen 19-9 (CA 19-9)], CT-reported results (tumour size, T stage and LN status), pathological characteristics of surgical specimens (histological type, tumour differentiation, T stage and LN status), the number of LNs harvested and follow-up data (follow-up duration and survival status), was collected. CEA, CA 19-9 and CT-reported results were obtained from the routine preoperative examination within 1 week before the surgery. Elevated CEA indicated a level of 5 ng/mL or greater, and elevated CA 19-9 indicated a level of 37 U/mL or greater. CT-reported tumour size was measured from the longest diameter. CT-reported T stage was evaluated according to the deformities of bowel wall and the appearance of the adjacent soft tissue. CT-reported LN status was determined based on the size, density and shape of the LNs. LNs with a diameter >10 mm, an irregular border or central necrosis, or formed a collection or grouped with a tendency to adhere to each other, were considered as positive CT-reported LN status. Tumour location was categorised as the colon and rectum. Tumour differentiation was categorised as well differentiated, moderately differentiated, poorly differentiated and undifferentiated. Histological type was categorised as adenocarcinoma, mucinous adenocarcinoma and signet-ring cell carcinoma. The follow-up duration was recorded from the time of surgery to the last follow-up date, and the information regarding the survival status at the last follow-up was collected. The pathological diagnosis of LN metastasis was determined on the basis of the harvested LNs.

### Assessment of the TSP

The corresponding 5-μm H&E-stained sections of the preoperative biopsies initially used for the diagnosis of each patient were retrieved from the pathological archive, and the TSP was assessed by two independent investigators blinded to the LN status for analysis. Any difference in opinion was resolved by discussion with a third investigator.

All slides were first scanned and digitised using the Aperio ImageScope (Leica Biosystems, CA, USA) with the ×20  objective. Then, a representative area showing the most invasive part at low magnification (×5  objective) was selected. Subsequently, a single area in which both stroma and tumour existed at high magnification (×10  objective) and tumour cells were present on all sides of the field was chosen. Despite some heterogeneity in the TSP among biopsy tissue blocks throughout the entire slide, the regions with the largest amount of stroma and the worst differentiation were selected as the representative object for analysis according to the previous study.^15^ Tissues that contained mucin or necrosis in the selected field were visually excluded. The interrater reliability was evaluated [*κ* = 0.866; 95% confidence interval (CI), 0.822–0.908].

**Fig. 1:**
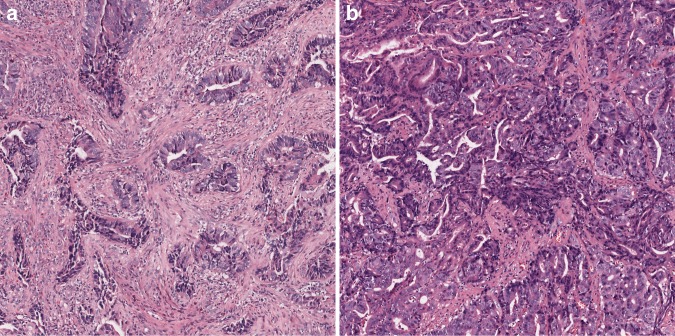
Example of TSP assessment in the preoperative biopsies of CRC. **a** High TSP ( > 50%). **b** Low TSP ( ≤ 50%).

The TSP was visually calculated (per tenfold: 10%, 20%, 30% and so forth) per field. For example, TSP 70% represented that stroma accounted for 70% of the entire tumour tissue, and tumour cells accounted for 30%. In this study, a TSP ≤50% was categorised as low TSP, and a TSP >50% was regarded as high TSP (Fig. 1).

### Association of TSP with LN metastasis

The association between TSP and LN metastasis was first assessed in both the training and validation cohorts. Stratified analyses were then performed to evaluate the differences in the LN status between high TSP and low TSP under each preoperative clinicopathological characteristics. Furthermore, the correlation of pathological T stage with TSP and the association of TSP with LN metastasis in different T stages were calculated. The relationship between TSP and LN metastasis in the negative CT-reported LN status (cN0) subgroup was also evaluated.

### Association of TSP with survival

The TSP in surgical specimens of CRC has been proved to be a prognostic biomarker;^13^ therefore, the association of the TSP in the preoperative biopsies with overall survival (OS) and disease-free survival (DFS) was evaluated.

### Development and evaluation of the nomogram

Univariate logistics regression analysis was conducted to assess the potential association of preoperative clinicopathological characteristics and TSP with the LN status in the training cohort. The characteristics with *P* < 0.05 were included in the multivariate logistic analysis. The backward stepwise selection was applied using Akaike’s information criteria as the stopping rule.^16^ A nomogram based on the multivariate logistic analysis was developed to calculate the individual risk of LN metastasis. The multicollinearity of the prediction model was evaluated using a variance inflation factor (VIF) and tolerance.^17^ The area under the receiver-operating characteristic curve (AUROC) was used to quantify the discriminative ability of the nomogram and each independent risk factor to predict LN metastasis. The calibration curve was plotted to graphically display the calibration of the nomogram, accompanied by the Hosmer–Lemeshow test.

### Validation of the nomogram

The bootstrap method was employed for internal validation in which the random samples drawn with a replacement from the original dataset were the same size as the training cohort. The 1000-bootstrap repetitions were performed, and the Harrell’s C-index was measured.^18^ The nomogram was then applied in the validation cohort for external validation, and the AUROC and calibration curve were also derived in the validation cohort.

### Clinical usefulness

A decision-curve analysis was performed to quantify the net benefits at different threshold probabilities to evaluate the clinical usefulness of the nomogram.^19^ The decision-curve analysis was a novel device for assessing the potential population impact of adopting a risk-prediction instrument into clinical practice. The context for decision-curve analysis was a situation in which individuals’ risks for an undesirable outcome were assessed, and individuals with sufficiently high risk were recommended for some intervention or treatment.^20^ The decision-curve analysis provided a net benefit, which was calculated using the following formula:

$${\mathrm{Net}}\;{\mathrm{benefit = true - positive}}\;{\mathrm{rate-false}} - {\mathrm{positive}}\;{\mathrm{rate}} \times \left[ {P_{\mathrm{t}}/\left( {1-P_{\mathrm{t}}} \right)} \right],$$

where *P*_t_ was the threshold probability where the expected benefit of treatment was equal to the expected benefit of avoiding treatment. In this study, *P*_t_ indicated the threshold probability of LN metastasis.

In addition, the maximum Youden index was selected as the cutoff value, and the patients were divided into high-risk and low-risk subgroups. The sensitivity, specificity, accuracy, positive predictive value (PPV) and negative predictive value (NPV) of the nomogram were calculated.

### Comparison with the clinicopathological nomogram

A clinicopathological nomogram based on the preoperative clinicopathological characteristics after a multivariate logistic analysis was constructed. The performance of the TSP-based nomogram was compared with that of the clinicopathological nomogram.

### Statistical analysis

Continuous variables were compared using an independent-sample, unpaired two-tailed *t* test or Mann−Whitney *H* test, as appropriate. Differences in categorical variables were compared using the chi-square or Fisher’s exact test. A logistic regression model was used to estimate the odds ratio (OR) and 95% CI and identify the independent predictors of LN metastasis. Survival curves were generated by using Kaplan–Meier survival analysis, and the differences in survival distributions were tested using the log-rank test. The Cox proportional hazard model was used to determine the hazard ratio (HR) of preoperative variables for OS and DFS. All statistical analyses were conducted using the R software (version 3.4.2) and SPSS (version 19.0), and a two-sided *P-*value  < 0.05 was considered as statistically significant.

## Results

### Patients

The clinicopathological characteristics of enrolled patients in this study are summarised in Table 1. The overall incidence of LN metastasis was 41.13% (218/530), with 47.28% (148/313) in the training cohort and 39.54% (70/177) in the validation cohort (*P* = 0.600). The distribution of preoperative tumour differentiation, CT-reported LN status and the number of harvested LNs between the training and validation cohorts, rather than other clinicopathological characteristics and TSP, were significantly different.

**Table 1:** Characteristics of the participants in the training and validation cohorts.

Variable	Training cohort		*P*	Validation cohort		*P*	P*
	High TSP (*N* = 133)	Low TSP (*N* = 220)		High TSP (*N* = 66)	Low TSP (*N* = 111)		
Age, median (IQR)	61 (51−68)	59 (51−65.75)	0.209	57 (47−64.25)	60 (50−68)	0.161	0.315
Sex, no. (%)							
Male	76 (57.1)	72 (64.5)	0.166	40 (60.6)	69 (62.2)	0.837	0.969
Female	57 (42.9)	41 (35.5)		26 (39.4)	42 (37.8)		
Tumour location, no. (%)							
Rectum	51 (38.3)	87 (39.5)	0.823	25 (37.9)	39 (35.1)	0.713	0.512
Colon	82 (61.7)	133 (60.5)		41 (62.1)	72 (64.9)		
Preoperative tumour differentiation, no. (%)							
Well	172 (78.2)	91 (68.4)	0.065	31 (47.0)	52 (46.8)	0.995	<0.001
Moderate	41 (18.6)	32 (24.1)		30 (45.5)	51 (45.9)		
Poor and undifferentiated	7 (3.2)	10 (7.5)		5 (7.6)	8 (7.2)		
Preoperative histological type, no. (%)							
Adenocarcinoma	128 (96.2)	218 (99.1)	0.109	64 (97.0)	109 (98.2)	0.630	0.478
Mucinous	5 (3.8)	2 (0.9)		2 (3.0)	2 (1.8)		
CT-reported tumour size, no. (%)							
≤4 cm	67 (50.4)	109 (49.5)	0.880	38 (57.6)	57 (51.4)	0.422	0.407
>4 cm	66 (49.6)	111 (50.5)		28 (42.4)	54 (48.6)		
CT-reported T stage, no. (%)							
T1 and T2	15 (11.3)	44 (20.0)	0.033	8 (12.1)	20 (18.0)	0.299	0.793
T3 and T4	118 (88.7)	176 (80.0)		58 (87.9)	91 (91.0)		
CT-reported LN status, no. (%)							
Negative	45 (33.8)	88 (40.0)	0.247	28 (42.4)	57 (51.4)	0.250	0.022
Positive	88 (66.2)	132 (60.0)		38 (57.6)	54 (48.6)		
CEA level, no. (%)							
Normal	87 (65.4)	153 (69.5)	0.420	44 (66.7)	77 (69.4)	0.708	0.931
Elevated	46 (34.6)	67 (30.5)		22 (33.3)	34 (30.6)		
CA 19-9 level, no. (%)							
Normal	105 (78.9)	188 (85.5)	0.115	56 (84.8)	93 (83.8)	0.851	0.731
Elevated	28 (21.1)	32 (14.5)		10 (15.2)	18 (16.2)		
Pathological T stage, no. (%)							
T1	4 (3.0)	13 (5.9)	0.017	1 (1.5)	6 (5.4)	0.036	0.631
T2	17 (12.8)	47 (21.4)		5 (7.6)	21 (18.9)		
T3	23 (17.3)	50 (22.7)		14 (21.2)	29 (26.1)		
T4	89 (66.9)	110 (50.0)		46 (69.7)	55 (49.5)		
LN metastasis, no. (%)							
No	36 (27.1)	169 (76.8)	<0.001	22 (33.3)	85 (76.6)	<0.001	0.600
Yes	97 (72.9)	51 (23.2)		44 (66.7)	26 (23.4)		
Postoperative tumour differentiation, no. (%)							
Well	9 (6.8)	32 (14.5)	0.138	3 (4.5)	12 (10.8)	0.347	0.253
Moderate	104 (78.2)	155 (70.5)		49 (74.2)	78 (70.3)		
Poor and undifferentiated	20 (15.0)	33 (15.0)		14 (21.2)	21 (18.9)		
Postoperative histological type, no. (%)							
Adenocarcinoma	121 (91.0)	197 (89.5)	0.663	62 (93.9)	102 (91.9)	0.309	0.051
Mucinous	12 (9.0)	23 (10.5)		4 (6.1)	7 (6.3)		
Signet-ring cell	0	0		0	2 (1.8)		
Number of harvested LNs, median (IQR)	26 (18−39)	26 (16.5−38)	0.673	20 (15–29)	21 (17–29)	0.951	<0.001

*CA* carbohydrate antigen, *CEA* carcinoembryonic antigen, *CT* computed tomography, *IQR* interquartile range, *LN* lymph node, *TSP* tumour stroma percentage.

P*, difference between the training and validation cohorts

### Association of TSP with LN metastasis

In the training cohort, 133 patients had high TSP and 220 had low TSP. High TSP was significantly associated with LN metastasis (*P* < 0.001), with an AUROC of 0.740 (95% CI: 0.694–0.786) in the training cohort (Table 1; Supplementary Fig. S2a). The subgroup analyses revealed that high TSP was still significantly associated with LN metastasis, regardless of any preoperative clinicopathological characteristics (Supplementary Fig. S3a). Similar results were obtained in the validation cohort (Supplementary Fig. S2b, S3b). In addition, TSP was significantly correlated with T stage in both the training and validation cohorts (*P* = 0.017 and 0.036, respectively). In the training cohort, 76.5% (13/17) of the T1 patients were low TSP, and 73.4% (47/64) of the T2 patients were low TSP. In T3 patients, this percentage decreased to 68.5% (50/73), and in T4 patients, it was only 55.3% (110/199) (Table 1; Supplementary Fig. S4a). The AUROC of TSP to LN metastasis in T1, T2, T3 and T4 was 0.837 (95% CI: 0.565−0.999), 0.740 (95% CI: 0.592−0.889), 0.763 (95% CI: 0.639−0.887) and 0.714 (0.641−0.787), respectively (Supplementary Fig. S4b). In the validation cohort, the percentages of low TSP in T1, T2, T3 and T4 were 85.7% (6/7), 80.8% (21/26), 67.4% (29/43) and 54.5% (55/101), respectively (Supplementary Fig. S4c), and the corresponding AUROC values of TSP to LN metastasis were 0.750 (95% CI: 0.260−0.999), 0.700 (95% CI: 0.431−0.969), 0.711 (95% CI: 0.539−0.882) and 0.691 (95% CI: 0.586−0.796) (Supplementary Fig. S4d). In the cN0 subgroup, 91 patients had negative LN metastasis on pathological evaluation, with 75 (82.4%) patients having low TSP in the training cohort. Furthermore, 42 patients had positive LN metastasis on pathological evaluation, with 29 (69.0%) patients having high TSP (*P* < 0.001) (Supplementary Fig. S5a). The AUROC of TSP to LN metastasis in patients with cN0 was 0.757 (95% CI: 0.663–0.851) (Supplementary Fig. S5b). In the validation cohort, 61 patients had negative LN metastasis on pathological evaluation in the cN0 subgroup, with 48 (78.7%) patients having low TSP. Moreover, 24 patients had negative LN metastasis on pathological evaluation, with 15 (62.5%) patients having high TSP (*P* < 0.001) (Supplementary Fig. S5c), and an AUROC of 0.706 (95% CI: 0.594−0.818) was found (Supplementary Fig. S5d).

### Association of TSP with survival

The median follow-up of the total cohort was 24 [interquartile range (IQR): 16–37] months. The estimated 3-year OS and DFS were 80.0% (95% CI: 75.5%−84.7%) and 71.4% (95% CI: 66.9%−76.1%), respectively (Supplementary Fig. S6).

The association of TSP with OS and DFS was analysed in the total cohort. In the high-TSP subgroup, the 3-year OS was 64.4% (95% CI: 56.3%−73.6%), compared with 89.7% (95% CI: 85.2%−94.5%) in the low-TSP subgroup (log-rank *P* < 0.001). For the DFS, the 3-year survival rates for the high-TSP and the low-TSP subgroups were 50.6% (95% CI: 43.0%−59.4%) and 84.4% (95% CI: 79.9%–89.3%), respectively (log-rank *P* < 0.001) (Supplementary Fig. S7). OS and DFS in the high-TSP subgroup were significantly lower than those in the low-TSP subgroup as expected, with HRs of 4.289 (95% CI: 2.624−7.008; *P* < 0.001) and 4.126 (95% CI: 2.841−5.993; *P* < 0.001), respectively, in the Cox regression analysis. The TSP was still an independent preoperative predictor of OS (HR: 3.977; 95% CI: 2.428−6.514; *P* < 0.001) and DFS (HR: 3.839; 95% CI: 2.639−5.584; *P* < 0.001) after adjusting for the preoperative clinicopathological factors (Supplementary Table S1).

### Development and assessment of the prediction model

**Fig. 2:**
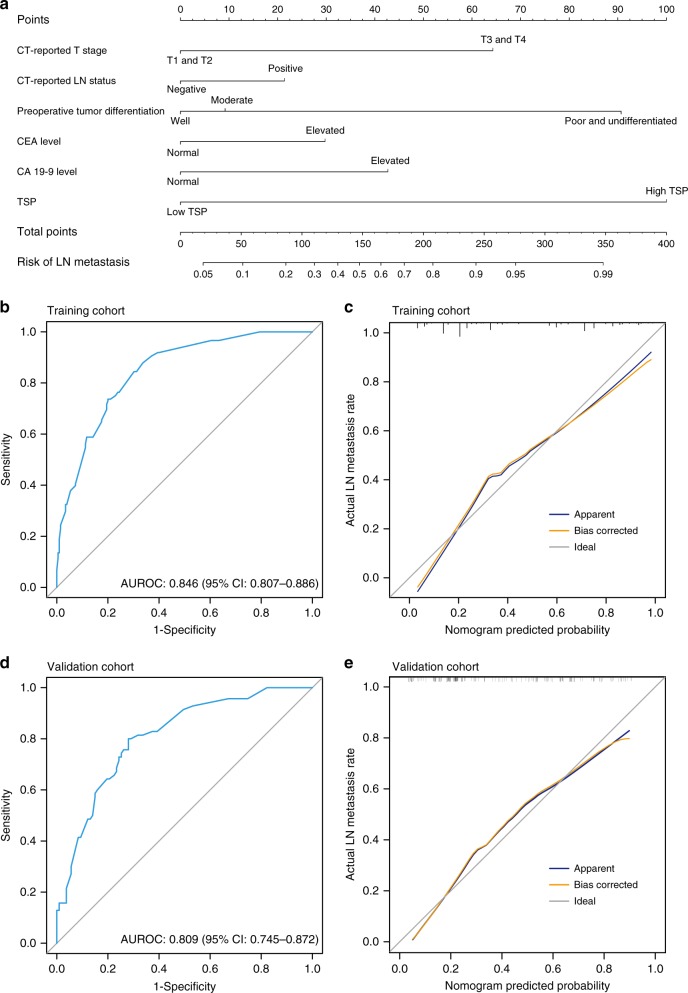
Nomogram and performance evaluation. **a** Newly developed TSP-based nomogram. **b** ROC curve of the nomogram in the training cohort. **c** Calibration curve of the nomogram in the training cohort. **d** ROC curve of the nomogram in the validation cohort. **e** Calibration curve of the nomogram in the validation cohort. In the calibration curve, the *y* axis represents the actual LN metastasis rate, and the *x* axis represents the nomogram-predicted LN metastasis probability. The diagonal grey line represents a perfect prediction using an ideal model. The blue line represents the performance of the nomogram. The orange line represents the bias-corrected performance of the nomogram.

In the training cohort, the univariate logistic analysis revealed that the CT-reported T stage, CT-reported LN status, preoperative tumour differentiation, CEA level, CA 19-9 level and TSP were statistically significant with LN metastasis in CRC (Table 2). The TSP showed the most predictive discrimination compared with the other five factors in both the training and validation cohorts (Supplementary Fig. S2). The multivariate logistic analysis indicated that the CT-reported T stage, CT-reported LN status, preoperative tumour differentiation, CEA level, CA 19-9 level and TSP were still associated with LN metastasis after backward stepwise selection. A prediction model was developed by incorporating the aforementioned six predictors, and a TSP-based nomogram was applied to provide the clinician a quantitative tool for evaluating the individual risk of LN metastasis (Fig. 2a). The VIF of each predictor was <10, and the corresponding tolerance was >0.1, indicating no multicollinearity in the prediction model (Supplementary Table S2).^17^ The AUROC of the nomogram in the training cohort was 0.846 (95% CI: 0.807−0.886) (Fig. 2b). The calibration curve showed that the nomogram-predicted LN metastasis probability was in good agreement with the observation in the training cohort, with a Hosmer–Lemeshow test *P*-value of 0.192 (Fig. 2c).

**Table 2:** Univariate and multivariate logistic analyses in the training cohort.

Variable	Univariate analysis	*P*	Multivariate analysis	*P*
	OR (95% CI)		OR (95% CI)	
Age	0.995 (0.798−1.013)	0.601	−	−
Sex (female vs. male)	1.438 (0.931−2.220)	0.101	−	−
Location (colon vs. rectum)	1.150 (0.745−1.777)	0.528	−	−
Preoperative histological type (mucinous vs. adenocarcinoma)	3.549 (0.679−18.548)	0.133	−	−
Preoperative tumour differentiation		0.002		0.036
Well	Reference	>0.99	Reference	>0.99
Moderate	1.551 (0.919−2.616)	0.1	1.234 (0.665−2.325)	0.515
Poor and undifferentiated	12.628 (2.828−56.391)	0.001	7.975 (1.608−39.537)	0.011
CT-reported tumour size (>4 cm vs. ≤ 4 cm)	1.037 (0.680−1.583)	0.865	−	−
CT-reported T stage (T3 and T4 vs. T1 and T2)	4.982 (2.363−10.502)	<0.001	4.358 (1.761−10.784)	0.001
CT-reported LN status (positive vs. negative)	2.015 (1.283−3.163)	0.002	1.632 (0.925−2.880)	0.091
CEA level (elevated vs. normal)	2.706 (1.709−4.284)	<0.001	1.976 (1.093−3.573)	0.024
CA 19-9 level (elevated vs. normal)	3.751 (2.072−6.792)	<0.001	2.658 (1.273−5.548)	0.009
TSP (high vs. low)	8.929 (5.446−14.638)	<0.001	9.872 (5.675−17.172)	<0.001

*CA* carbohydrate antigen, *CEA* carcinoembryonic antigen, *CI* confidence interval, *CT* computed tomography, *LN* lymph node, *OR* odds ratio, *TSP* tumour stroma percentage

### Prediction model validation

After employing the bootstrap method with 1000-bootstrap repetitions in the training cohort, the results remained largely unchanged between iterations, with a mean C-index of 0.834. Furthermore, favourable discrimination of the prediction model was validated in the validation cohort, with an AUROC of 0.809 (95% CI: 0.745−0.872) (Fig. 2d). Also, good calibration was found, and the Hosmer–Lemeshow test demonstrated no statistically significant difference (*P* = 0.356) (Fig. 2e).

### Clinical usefulness

**Fig. 3:**
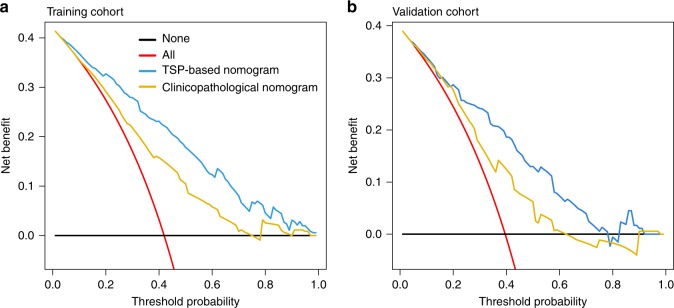
Decision-curve analysis. **a** Decision curve in the training cohort. **b** Decision curve in the validation cohort. The red line and black line represent the assumption regarding all patients with and without LN metastasis, respectively. The blue line represents the TSP-based nomogram, and the orange line represents the clinicopathological nomogram.

The decision-curve analysis of the TSP-based nomogram in the training and validation cohorts is shown in Fig. 3. The *x* axis indicated the threshold probability, and the *y* axis indicated the net benefit. The black line represented the assumption that no patient had LN metastasis, and the red line represented the assumption that all patients had LN metastasis. The decision-curve analysis demonstrated that using the TSP-based nomogram to detect the LN status could add more net benefit compared with the treating-all-patients scheme or treating-none scheme.

The maximum Youden index of 0.310 was selected as the cutoff value of the nomogram in the training cohort, and all patients were divided into high-risk and low-risk subgroups. The sensitivity, specificity, accuracy, NPV and PPV of the nomogram were 84.5%, 69.8%, 75.9%, 86.1% and 66.8% in the training cohort, respectively. In the validation cohort, a sensitivity of 80.0%, a specificity of 72.0%, an accuracy of 75.1%, an NPV of 84.6% and a PPV of 65.1% were found. In all 530 patients, a sensitivity of 83.0%, a specificity of 70.5%, an accuracy of 75.7%, an NPV of 85.6% and a PPV of 66.3% were also detected (Supplementary Table S3).

### Performance of the nomogram in different T stages

The nomogram-predicted high-risk and low-risk subgroups correlated with different T stages in the training, validation and total cohorts (Supplementary Fig. S8a−c). A higher percentage of high-risk patients was significantly related to the advanced T stage (*P* < 0.001) (Supplementary Table S4). The performance of the nomogram to predict LN metastasis in different T stages was evaluated. For all T1 patients, the diagnostic accuracy was 87.5%, with a sensitivity of 50%, a specificity of 100%, an NPV of 85.7% and a PPV of 100%, and the nomogram yielded an AUROC of 0.917 (95% CI: 0.802−0.999), indicating that the nomogram had favourable discrimination in T1 patients. In terms of all T2 patients, a sensitivity of 70.8%, a specificity of 84.9%, an accuracy of 81.1%, an NPV of 88.9% and a PPV of 63.0% were achieved, and good discrimination was observed (AUROC: 0.843; 95% CI: 0.756−0.930). Meanwhile, a sensitivity of 80.5%, a specificity of 70.7%, an accuracy of 74.1%, an NPV of 86.9% and a PPV of 60.0% were discovered in all T3 patients, with an AUROC of 0.826 (95% CI: 0.747−0.904). Furthermore, the overall sensitivity, specificity, accuracy, NPV and PPV of the nomogram were 87.1%, 60.8%, 73.7%, 83.2% and 68.1% in all T4 patients, respectively, and the good discriminatory ability was also found (AUROC: 0.818; 95% CI: 0.772−0.865). Similar findings were confirmed in both the training and validation cohorts (Supplementary Fig. S8d−f, Supplementary Table S3).

### Performance of the nomogram in the cN0 subgroup

The performance of the TSP-based nomogram to predict LN metastasis in the cN0 subgroup was assessed. In the training cohort, 83.5% (76/91) patients were assigned to the nomogram-predicted low-risk subgroup in pathologically diagnosed negative LN metastasis, and 71.4% (30/42) were assigned to the high-risk subgroup in pathologically diagnosed positive LN metastasis (*P* < 0.001) (Supplementary Fig. S9a, Supplementary Table S4). The AUROC to predict LN metastasis was 0.853 (95% CI: 0.787−0.918) (Supplementary Fig. S9b), with the sensitivity, specificity, accuracy, NPV and PPV of 71.4%, 83.5%, 79.7%, 86.4% and 66.7%, respectively (Supplementary Table S3). In the validation cohort, a sensitivity of 79.2%, a specificity of 73.8%, an accuracy of 75.3%, an NPV of 90.0% and a PPV of 54.3% were found for the prediction of LN metastasis in the cN0 subgroup, with 73.8% (45/61) patients predicted to have low risk of pathologically diagnosed negative LN metastasis and 79.2% (19/24) patients predicted to have high risk of pathologically diagnosed positive LN metastasis (*P* < 0.001) (Supplementary Fig. S9c). The AUROC was 0.785 (95% CI: 0.681−0.890) (Supplementary Fig. S9d). In total, the TSP-based nomogram showed a satisfactory ability to divide all cN0 patients into high-risk and low-risk subgroups (*P* < 0.001) (Supplementary Fig. S9e). The overall sensitivity, specificity, accuracy, NPV and PPV of the nomogram to predict LN metastasis were 74.2%, 79.6%, 78.0%, 87.8% and 61.0%, respectively, with an AUROC of 0.827 (95% CI: 0.771−0.883) (Supplementary Fig. S9f).

### Comparison with the clinicopathological nomogram

A clinicopathological nomogram, including CT-reported T stage, CT-reported LN status, preoperative tumour differentiation, CEA level and CA 19-9 level, was constructed after univariate and multivariate logistic analyses (Supplementary Table S5 and Supplementary Fig. S10a). The AUROC values of the clinicopathological nomogram in the training and validation cohorts was 0.735 (95% CI: 0.683−0.786) and 0.698 (95% CI: 0.621−0.774), respectively (Supplementary Figs. S10b, S10c).

Compared with the clinicopathological nomogram, the TSP-based nomogram, in which TSP was added to the clinicopathological nomogram, displayed significantly improved performance in the training, validation and total cohorts (Table 3; Supplementary Fig. S11). In addition, the decision-curve analysis indicated that the TSP-based nomogram had a higher net benefit compared with the clinicopathological nomogram across the majority of the range of threshold probabilities in both the training and validation cohorts (Fig. 3).

**Table 3:** Performance comparison between TSP-based and clinicopathological nomogram.

Models	AUROC (95% CI)	*P*
Training cohort		
TSP-based nomogram	0.846 (0.807–0.886)	<0.001
Clinicopathological nomogram	0.735 (0.683–0.786)	
Validation cohort		
TSP-based nomogram	0.809 (0.745–0.872)	0.002
Clinicopathological nomogram	0.698 (0.621–0.774)	
Total cohort		
TSP-based nomogram	0.830 (0.800–0.859)	<0.001
Clinicopathological nomogram	0.721 (0.684–0.758)	

*AUROC* area under receiver-operating characteristic curve, *CI* confidence interval, *TSP* tumour stroma percentage

## Discussion

Accurate assessment of the LN status before surgery is needed for tailored therapy in CRC. This study investigated the role of TSP of the preoperative biopsies in predicting the LN status in CRC. A high TSP was significantly associated with LN metastasis in both the training and validation cohorts. Furthermore, a non-invasive diagnostic nomogram incorporating CT-reported T stage, CT-reported LN status, preoperative tumour differentiation, CEA level, CA 19-9 level and TSP was developed and validated, which predicted the risk of LN metastasis individually, and shows a good agreement between the predictive and actual LN metastasis probability. In addition, the decision-curve analysis revealed that more net benefit was added from the nomogram than the treating-all-patient or treating-none scheme.

Compared with the clinicopathological nomogram, which comprised CT-reported T stage, CT-reported LN status, preoperative tumour differentiation, CEA level and CA 19-9 level, the TSP-based nomogram showed more robust performance to predict the LN status (AUROC comparison: 0.830 vs. 0.721; *P* < 0.001). In addition, the decision-curve analysis also demonstrated that more net benefit would be achieved with the assistance of a TSP-based nomogram in estimating the risk of LN metastasis for decision-making. Therefore, the TSP-based nomogram was more robust in the individual diagnosis of LN metastasis compared with the clinicopathological nomogram in CRC.

The accuracy of CT for the preoperative prediction of the LN status was limited in CRC,^21^ and the overall accuracy was not >60%, despite combining with other image modalities, which was as accurate as flipping a coin.^7^ Inadequate nodal staging might oftentimes result in the under- or overtreatment of CRC, especially in T1 and T2 patients.^7^ The overall accuracy of the TSP-based nomogram was 74.3% under the optimal cutoff value. Notably, in T1 and T2 patients, the TSP-based nomogram showed an improved accuracy of 87.5% and 81.1%, respectively, with the AUROC values of 0.917 and 0.843 observed, which would help in the clinical decision-making regarding the therapy of CRC. In addition, patients diagnosed as cN0 were typically considered to be at low risk of LN metastasis. However, some cN0 patients still suffered from LN metastasis after the surgery. When patients were categorised into high- and low-risk subgroups, the high-risk subgroup had a significantly greater probability of having LN metastasis. The proposed TSP-based nomogram yielded a favourable accuracy of 78.0% and discrimination of 0.827 to identify actually patients with a high risk of LN metastasis in the cN0 subgroup.

In this study, TSP was assessed with the H&E-stained sections of the preoperative biopsies initially used for diagnosis. The high interrater reliability (*κ* = 0.866) indicated that TSP was a reproducible measurement for the preoperative CRC biopsies. Although several studies reported that the evaluation of TSP was performed on the most invasive part of surgical specimens, West et al.^22^ found that TSP on the luminal surface of CRC was also an independent predictor of survival. They held the view that TSP could potentially be evaluated on preoperative biopsies of CRC, and their findings allowed a further investigation to determine whether the results might be extrapolated in the diagnostic biopsy material. This study proved that it was feasible to evaluate TSP in the preoperative biopsies of CRC, and the TSP was significantly associated with LN metastasis and oncological outcomes. In addition, Yanagisawa et al.^14^ found that it was practicable to assess the stromal grade (similar to TSP) using needle biopsies of prostate cancer. Courrech et al. also demonstrated that it was feasible and reproducible to score TSP on oesophageal adenocarcinoma biopsies, and TSP was also independently associated with the survival of patients with oesophageal adenocarcinoma.^15^ Thus, although the proposed method using preoperative biopsies was different from the conventional method, high reproducibility and predictive impact of TSP in preoperative biopsies might promote its clinical translation in the era of precision medicine.

TSP was reported to be a prognostic biomarker in several types of cancer, and high TSP was associated with metastasis and worse survival. The underlying mechanisms for this observation were still unclear. However, the interactions between tumour cells and the surrounding host stroma influenced the initiation, progression, metastasis and patients’ prognosis.^23^ The coexistence of host stroma and tumour cells could maintain tumour growth and proliferation by inhibiting apoptosis and promote neovascularisation by the cooperation of various types of cells in the tumour microenvironment; otherwise, tumours would become dormant.^24,25^ In addition, as the major component of tumour stroma, accumulated cancer-associated fibroblasts were found to promote LN metastasis in oesophageal cancer.^26^ Meanwhile, increased collagen density at the tumour–stromal interface could also facilitate the local invasion of cancer cells.^27^ The results of this study revealed the relationship between TSP in the preoperative biopsies and LN metastasis in CRC, and future studies should focus on the investigation of the relevant molecular mechanisms.

Several investigators postulated some prediction models to predict the LN status of CRC preoperatively. Huang et al.^28^ presented a radiomics nomogram, incorporating the radiomics signature from CT and other risk factors, such as CT-reported LN status and CEA, with an AUROC value of 0.736. Qu et al.^29^ reported that a four-miRNA panel from serum samples, including miR-122-5p, miR-146b-5p, miR-186-5p and miR-193a-5p, combined with the CT-reported LN status, could also predict LN metastasis of CRC. However, the additional technical requirements are not accessible at most pathological or radiological departments, and the extraction and quantification of miRNA from serum samples place more economic burden on patients, thus limiting their clinical application. Still, these biomarkers, including TSP, radiomics signature and miRNA panel, can be used together to improve the accuracy for predicting LN metastasis of CRC preoperatively in the near future.

Indeed, compared with other potential biomarkers, incorporating the TSP assessment into clinical practice had some advantages. First, the TSP could be assessed on standard H&E-stained sections and observed by optical microscopy during the pathological diagnosis, hence not imposing additional costs on patients. Second, the TSP assessment took a minimal time (<2 min) and pathologists could conduct the TSP assessment without special training. Therefore, the implementation of this method in daily practice is a practical option. It was convenient to calculate the individual risk of LN metastasis because the CT-reported T stage, CT-reported LN status, preoperative tumour differentiation, CEA level and CA 19-9 level were acquired in routine clinical practice, and the TSP could be obtained during the pathological diagnosis.

This study had some limitations. First, it was a retrospective study, and hence not free from potential selection bias. Thus, a prospective clinical trial is needed to confirm the performance of the TSP-based nomogram. Second, all enrolled participants came from a single institution with limited generalisability. Therefore, cohorts from other institutions, especially from Western countries, are required to further validate the findings further.

In conclusion, this study indicated that the TSP in the preoperative biopsies was an independent predictor of LN metastasis in patients with CRC. The TSP-based nomogram, which incorporated the TSP in preoperative biopsies and other clinicopathological risk factors, could be used to evaluate the preoperative individualised risk of LN metastasis in CRC conveniently.

## Supplementary information

Revised supplementary material

## Data Availability

All data sets generated and/or analysed during this study are available from the corresponding author on reasonable request.
