# Supplementary material for: Association of the tumour stroma percentage in the preoperative biopsies with lymph node metastasis in colorectal cancer
Source: Br J Cancer. 2019 Dec 2;122(3):388–96. doi: 10.1038/s41416-019-0671-7 (PMC7000705; doi:10.1038/s41416-019-0671-7)
Supplement: Supplementary file 1 — Revised supplementary material [file 41416_2019_671_MOESM1_ESM.docx]

**Supplementary material**

**Table of Contents**

[**Supplementary Figures** 2](#_Toc22895064)

[**Fig. S1.** Recruitment pathway of patients in this study. 2](#_Toc22895065)

[**Fig. S2.** Discriminative ability of the TSP and other five independent risk clinicopathologic characteristics for the LN status. 3](#_Toc22895066)

[**Fig. S3.** Association of TSP with LN metastasis under each preoperative clinicopathological characteristic. 4](#_Toc22895067)

[**Fig. S4.** Subgroup analyses of TSP in different T stages. 5](#_Toc22895068)

[**Fig. S5.** Subgroup analyses of TSP in cN0 subgroup. 6](#_Toc22895069)

[**Fig. S6.** Kaplan−Meier survival analysis in all patients. 7](#_Toc22895070)

[**Fig. S7.** Kaplan-Meier analysis of overall survival and disease-free survival according to the TSP subgroups in all patients. 8](#_Toc22895071)

[**Fig. S8.** Subgroup analyses of nomogram-predicted high-risk and low-risk groups in different T stages. 9](#_Toc22895072)

[**Fig. S9.** Subgroup analyses of nomogram-predicted high-risk and low-risk groups in the cN0 subgroup. 10](#_Toc22895073)

[**Fig. S10.** Clinicopathological nomogram and its performance. 12](#_Toc22895074)

[**Fig. S11.** Performance comparison between TSP-based nomogram and clinicopathological nomogram in all patients. 13](#_Toc22895075)

[**Supplementary Tables** 14](#_Toc22895076)

[**Table S1.** Univariate and multivariate Cox regression analyses of the preoperative predictors for OS and DFS 14](#_Toc22895077)

[**Table S2.** Multicollinearity assessment of the TSP-based prediction model 15](#_Toc22895078)

[**Table S3.** Model performance in estimating the risk of LN status in different status 16](#_Toc22895079)

[**Table S4.** Distribution of nomogram-predicted LN risk in T stage and cN0 subgroup 17](#_Toc22895080)

[**Table S5.** Univariate and multivariate logistic analyses in the training cohort without TSP 18](#_Toc22895081)

**Supplementary Figures**

**
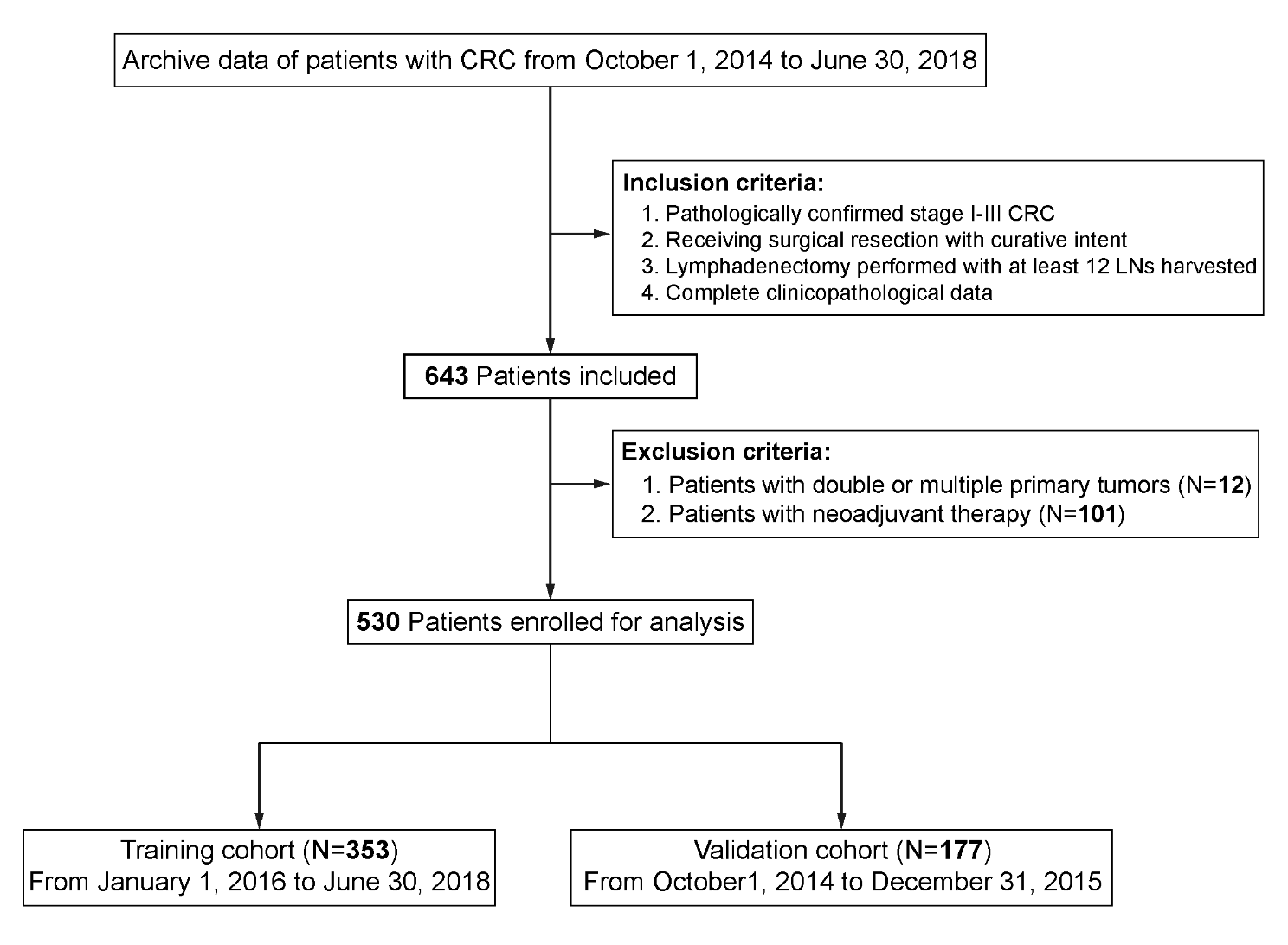
**

**Fig. S1.** Recruitment pathway of patients in this study.

**
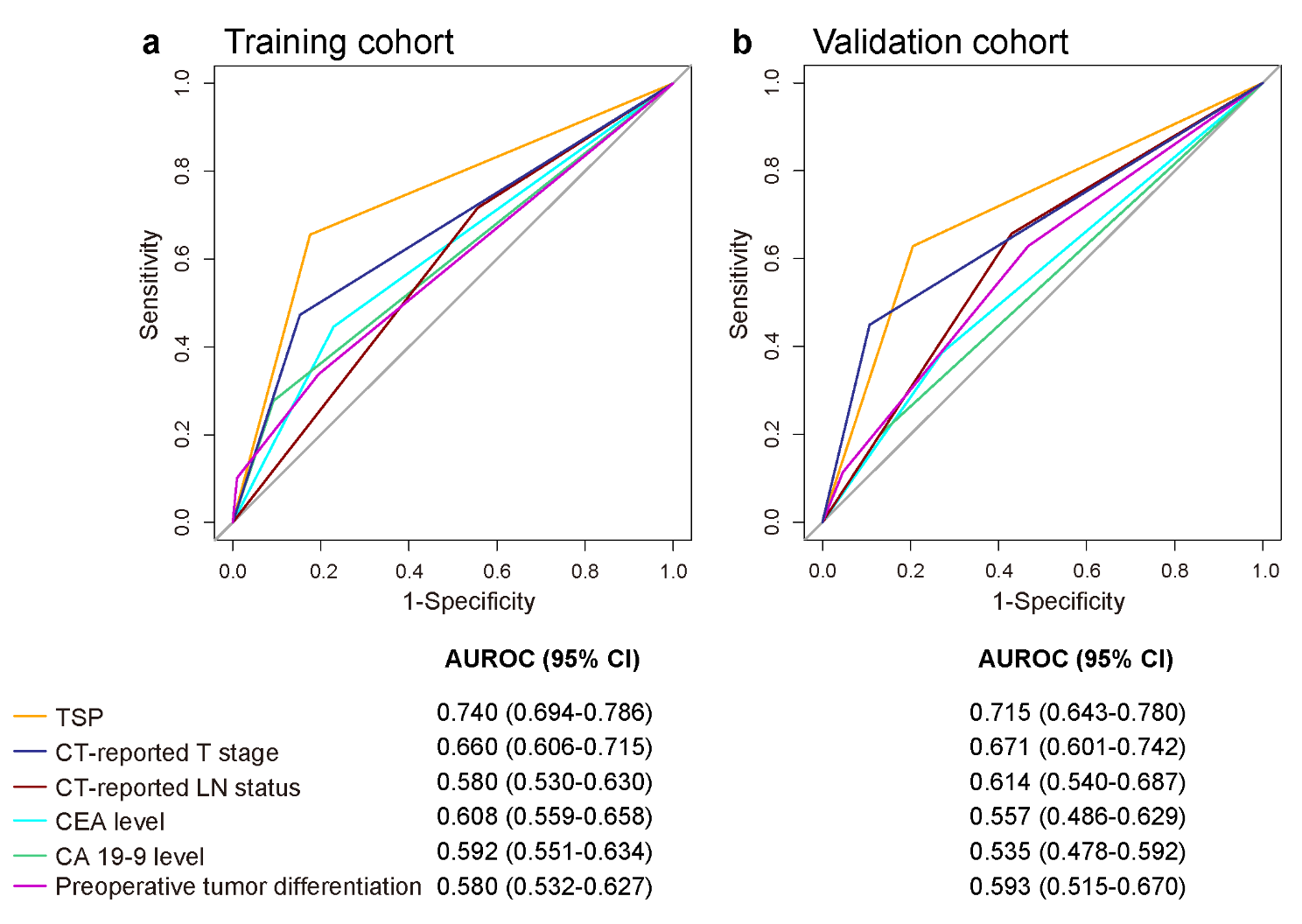
**

**Fig. S2.** Discriminative ability of the TSP and other five independent risk clinicopathologic characteristics for the LN status in the **(a)** training and **(b)** validation cohorts.

**
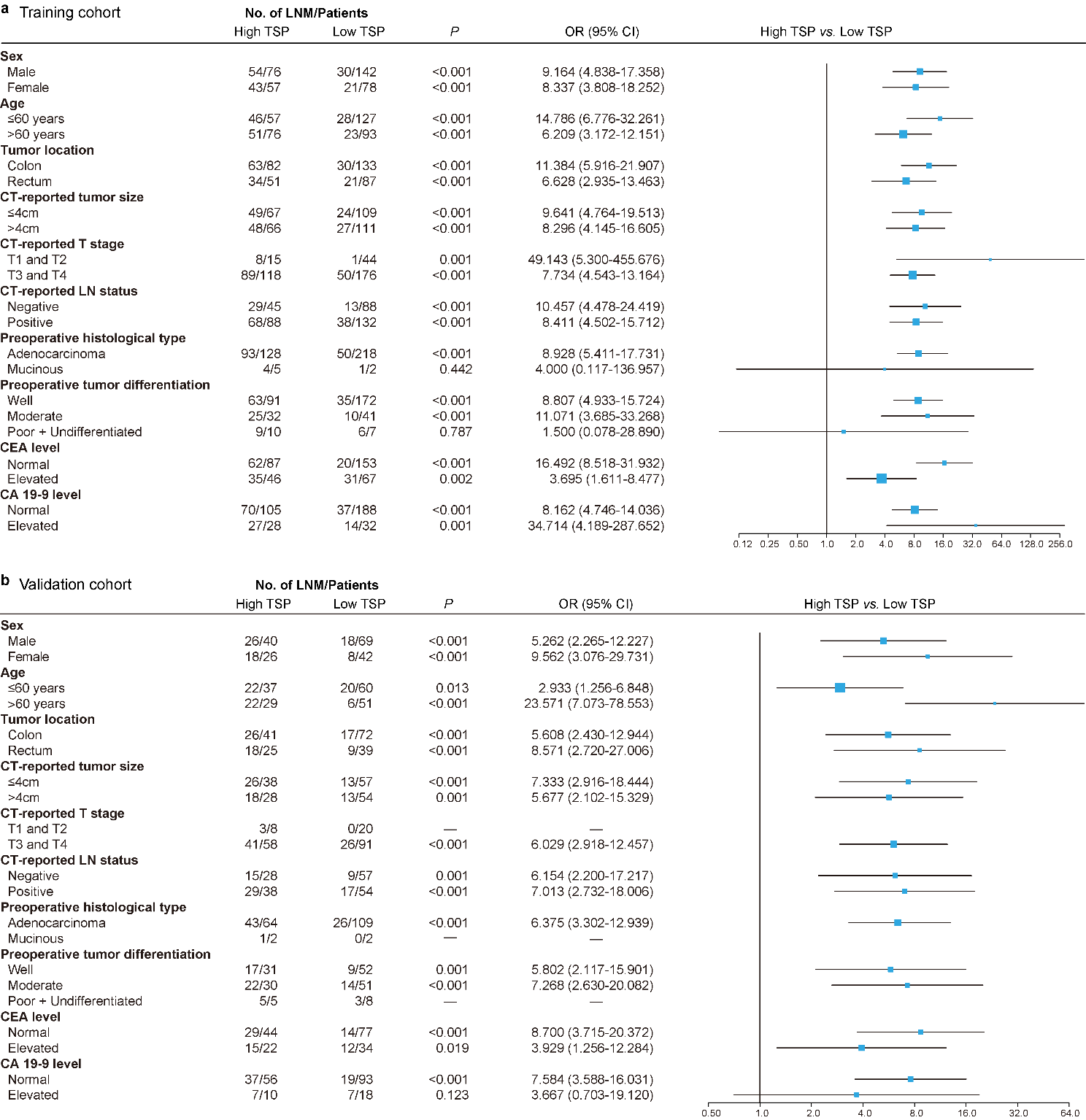
**

**Fig. S3.** Association of TSP with LN metastasis under each preoperative clinicopathological characteristic in the **(a)** training and **(b)** validation cohorts.

**
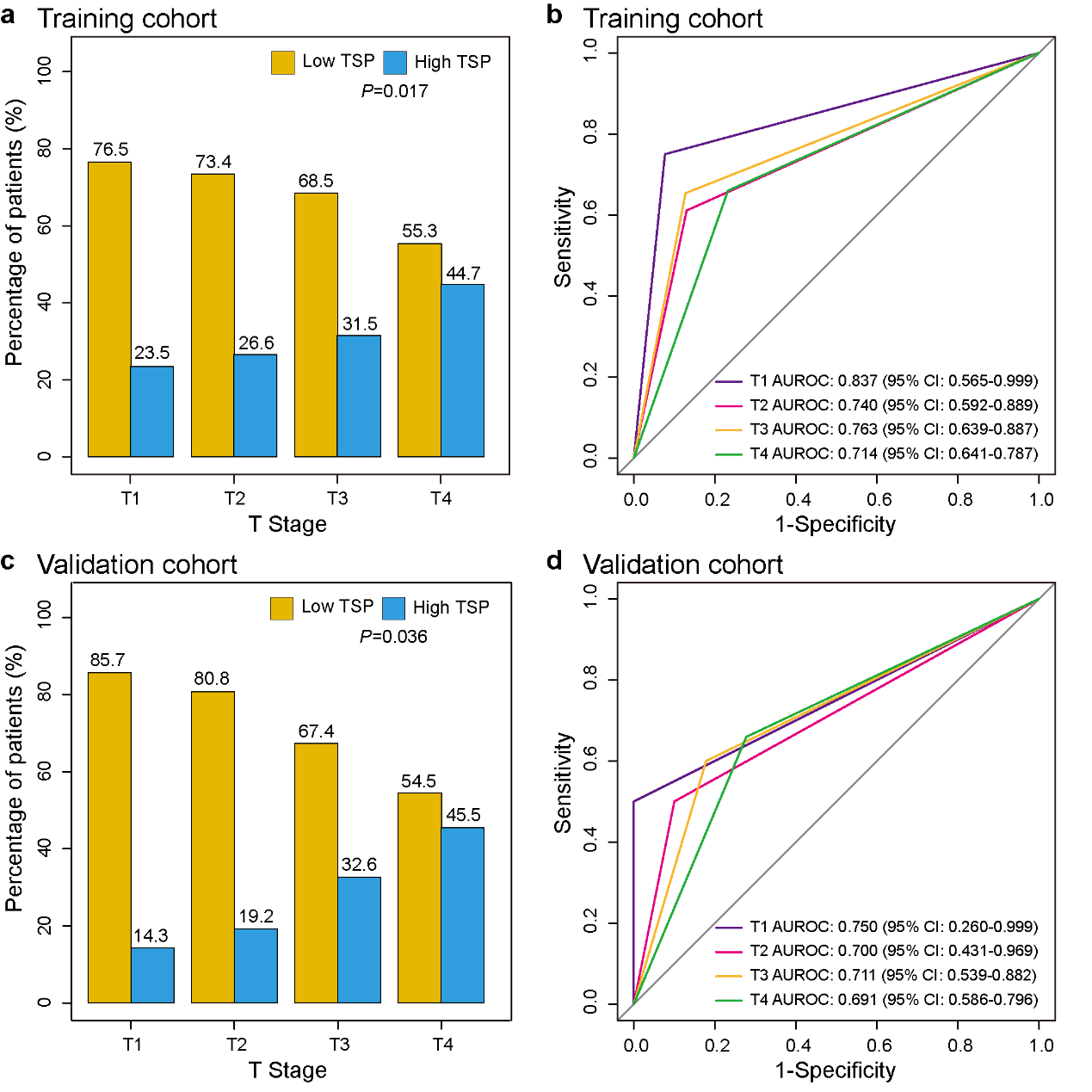
**

**Fig. S4.** Subgroup analyses of TSP in different T stages. **(a)** Correlation of T stage with the TSP in training cohort. **(b)** Performance of the TSP to predict LN metastasis in different T stages of the training cohort. **(c)** Correlation of T stage with the TSP in the validation cohort. **(d)** Performance of the TSP to predict LN metastasis in different T stages of the validation cohort.

**
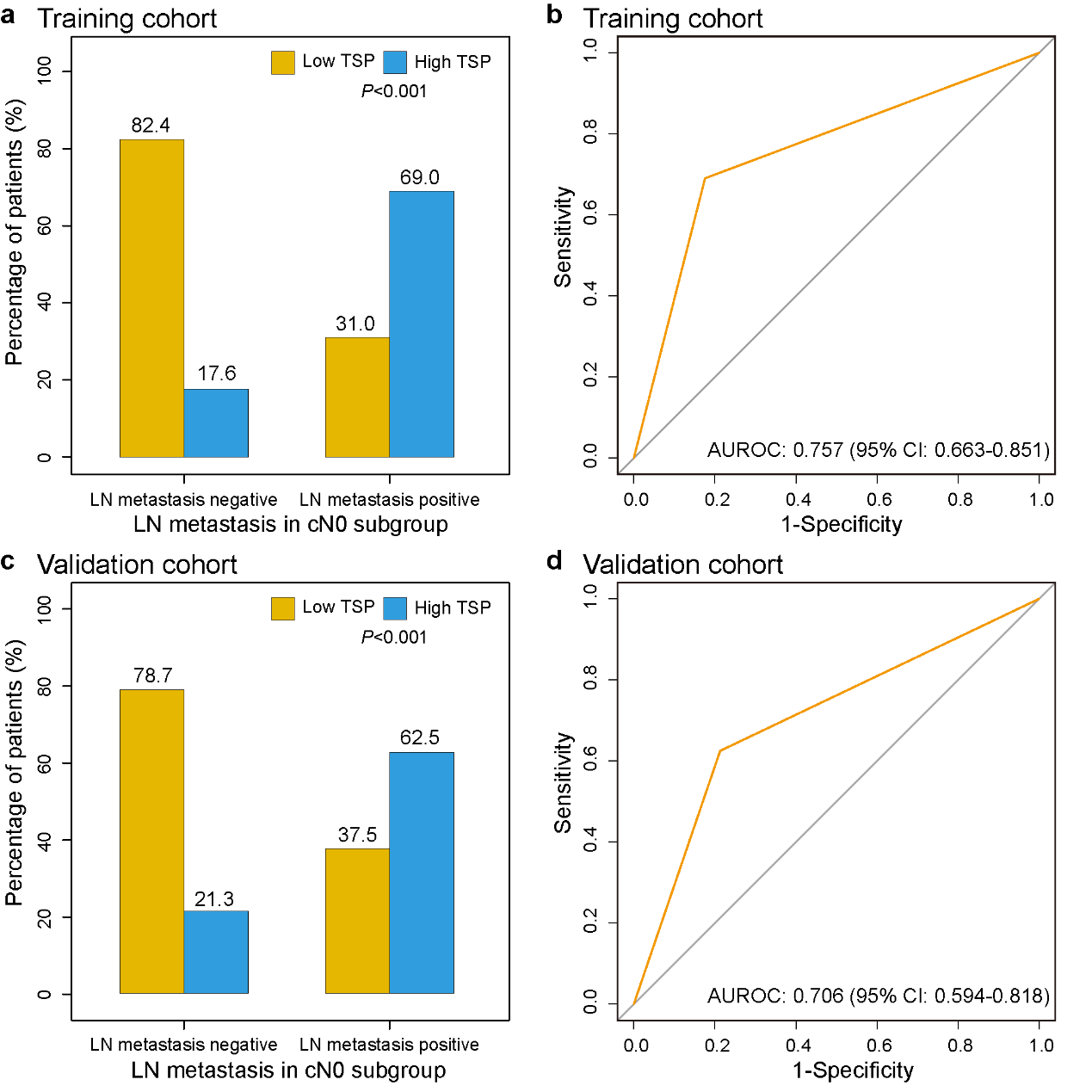
**

**Fig. S5.** Subgroup analyses of TSP in cN0 subgroup. **(a)** Correlation of LN metastasis status with the TSP in cN0 subgroup of the training cohort. **(b)** Performance of the TSP to predict LN metastasis in cN0 subgroup of the training cohort. **(c)** Correlation of LN metastasis status with the TSP in cN0 subgroup of the validation cohort. **(d)** Performance of the TSP to predict LN metastasis in cN0 subgroup of the validation cohort.

**
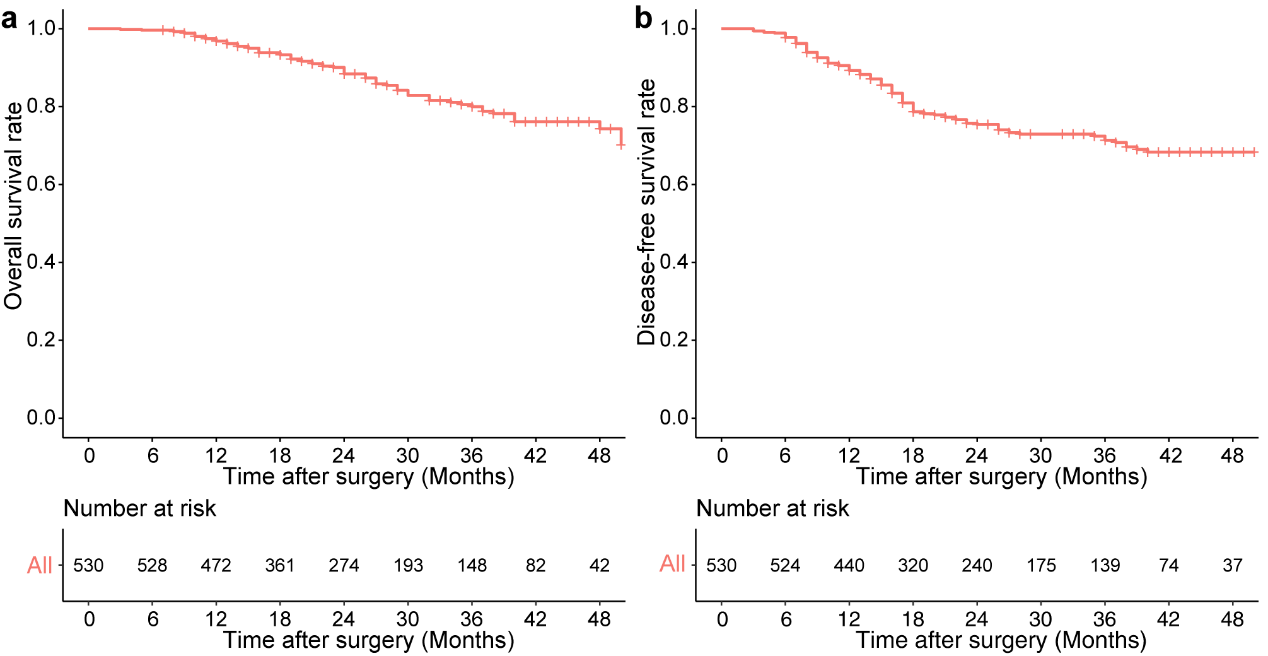
**

**Fig. S6.** Kaplan−Meier survival analysis in all patients. **(a)** Overall survival of all patients. **(b)** Disease-free survival of all patients.

**
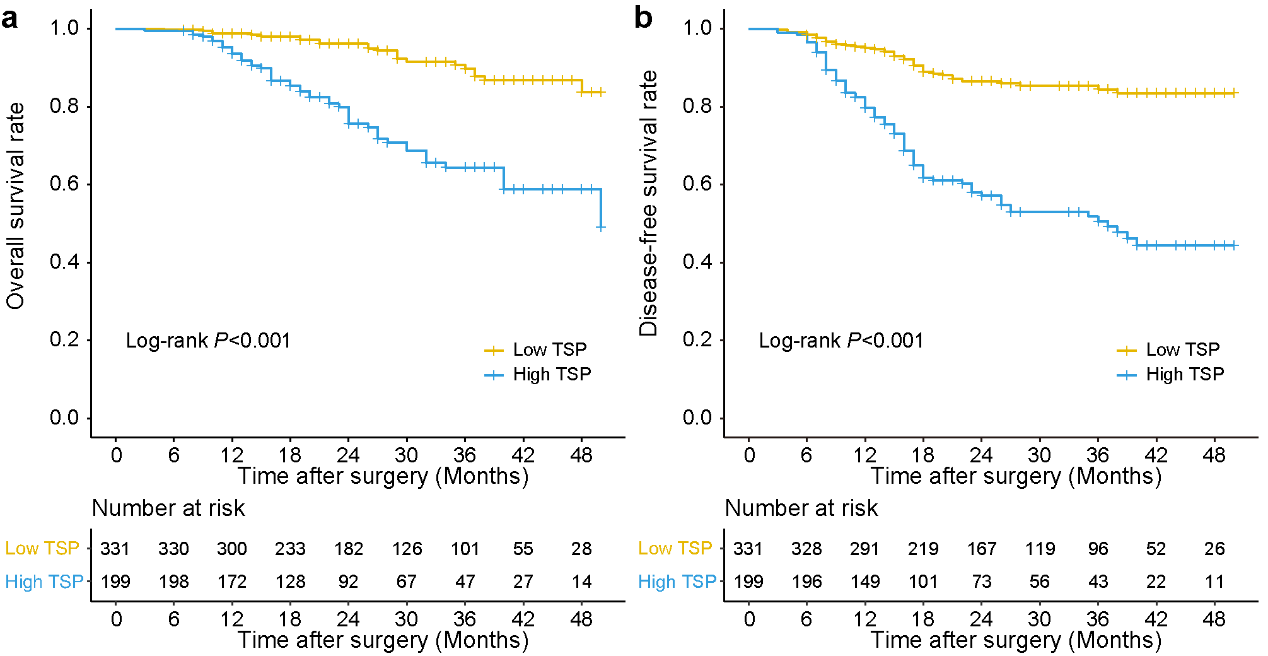
**

**Fig. S7.** Kaplan-Meier analysis of overall survival and disease-free survival according to the TSP subgroups in all patients. **(a)** Overall survival of all patients in the high- and low-TSP subgroups. **(b)** Disease-free survival of all patients in the high- and low-TSP subgroups.

**
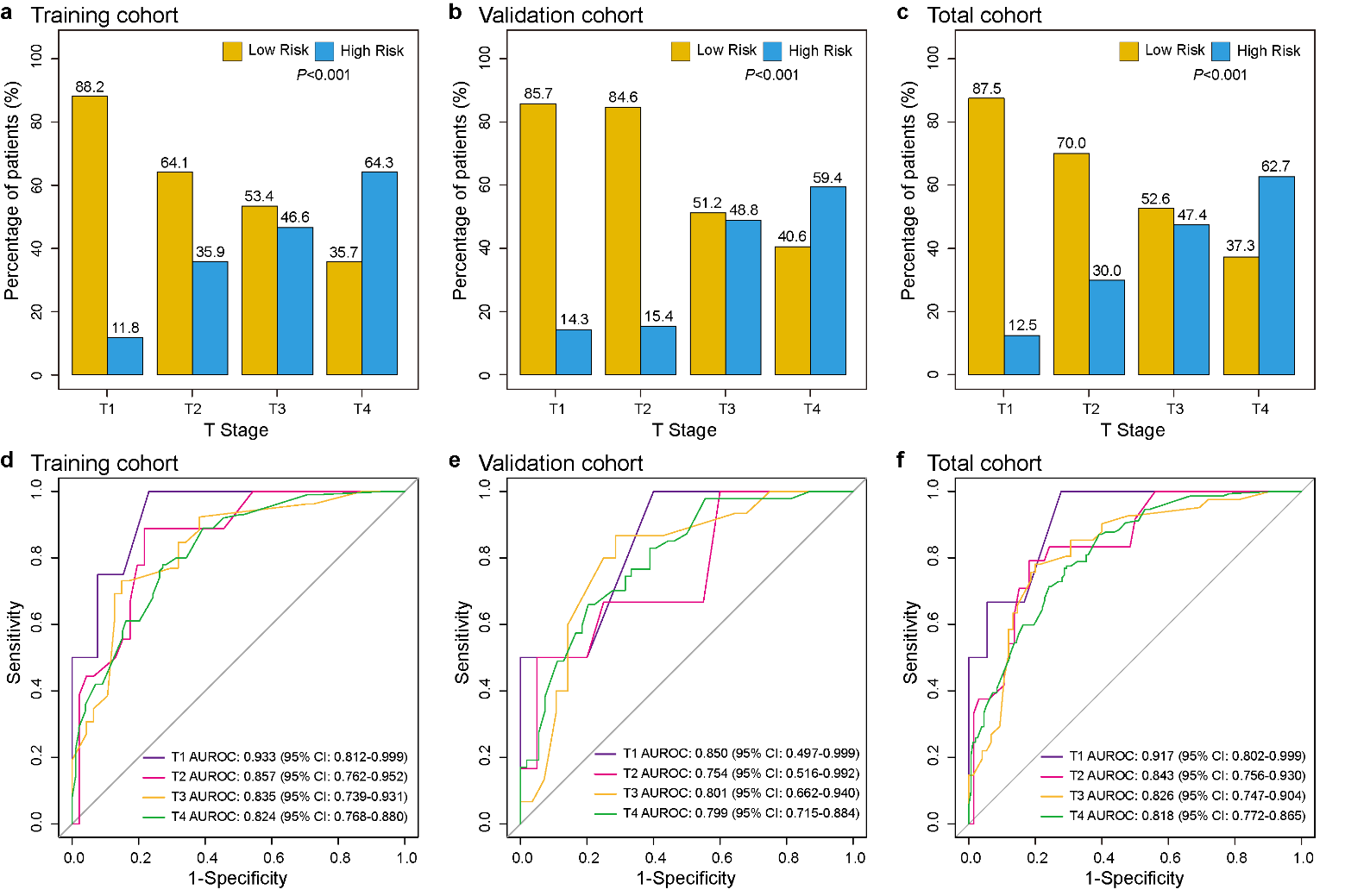
Fig. S8.** Subgroup analyses of nomogram-predicted high-risk and low-risk groups in different T stages. **(a−c)** Distribution of high-risk and low-risk patients in different T stages of the training, validation, and total cohorts. **(d−f)** Performance of the nomogram to predict LN metastasis in different T stages of the training, validation, and total cohorts.

**
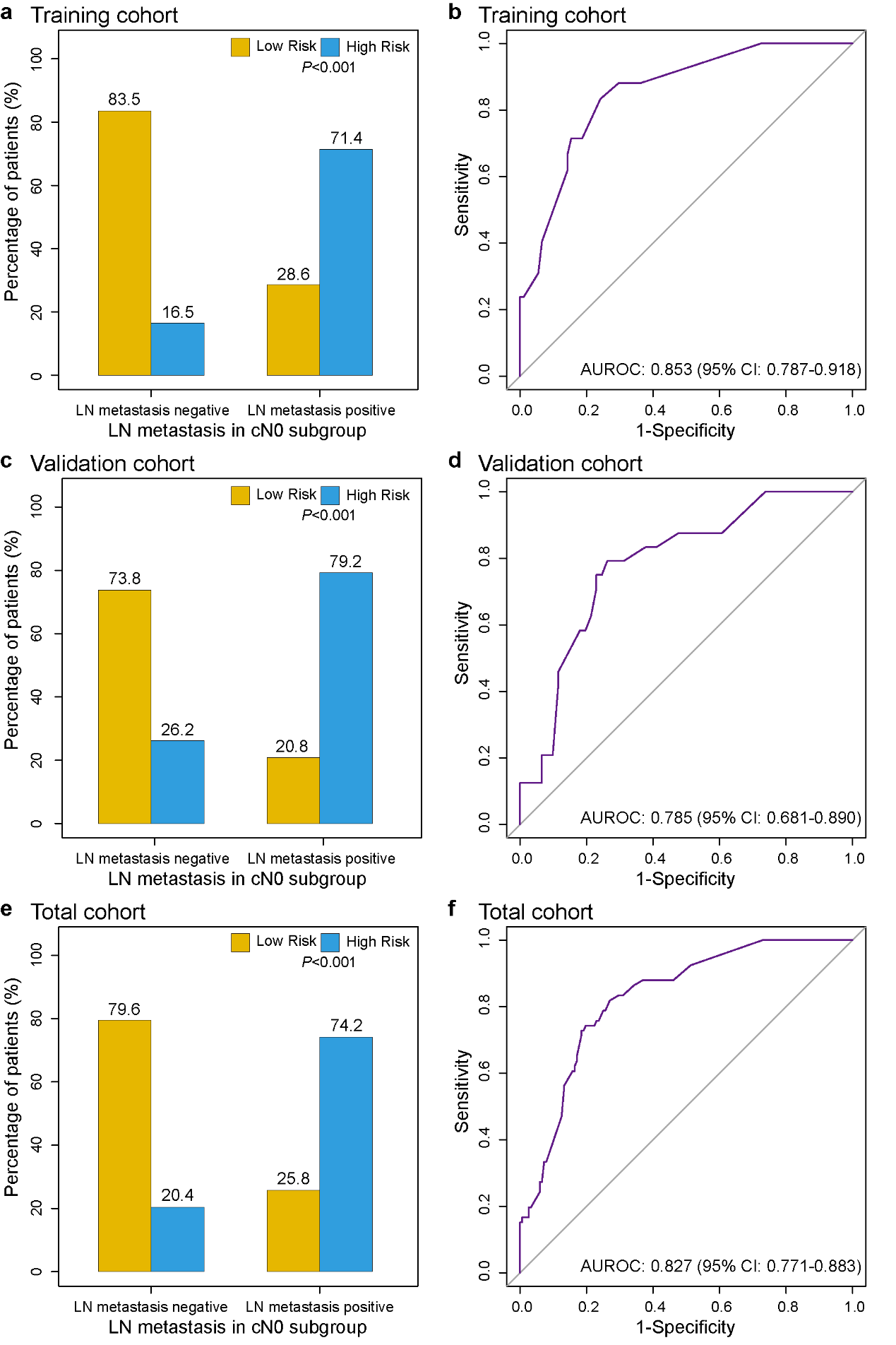
**

**Fig. S9.** Subgroup analyses of nomogram-predicted high-risk and low-risk groups in the cN0 subgroup. **(a)** Distribution of high-risk and low-risk patients in the cN0 subgroup of the training cohort. **(b)** Performance of the nomogram to predict LN metastasis in the cN0 subgroup of the training cohort. **(c)** Distribution of high-risk and low-risk patients in the cN0 subgroup of the validation cohort. **(d)** Performance of the nomogram to predict LN metastasis in the cN0 subgroup of the validation cohort. **(e)** Distribution of high-risk and low-risk patients in the cN0 subgroup of the total cohort. **(f)** Performance of the nomogram to predict LN metastasis in the cN0 subgroup of the total cohort.

**
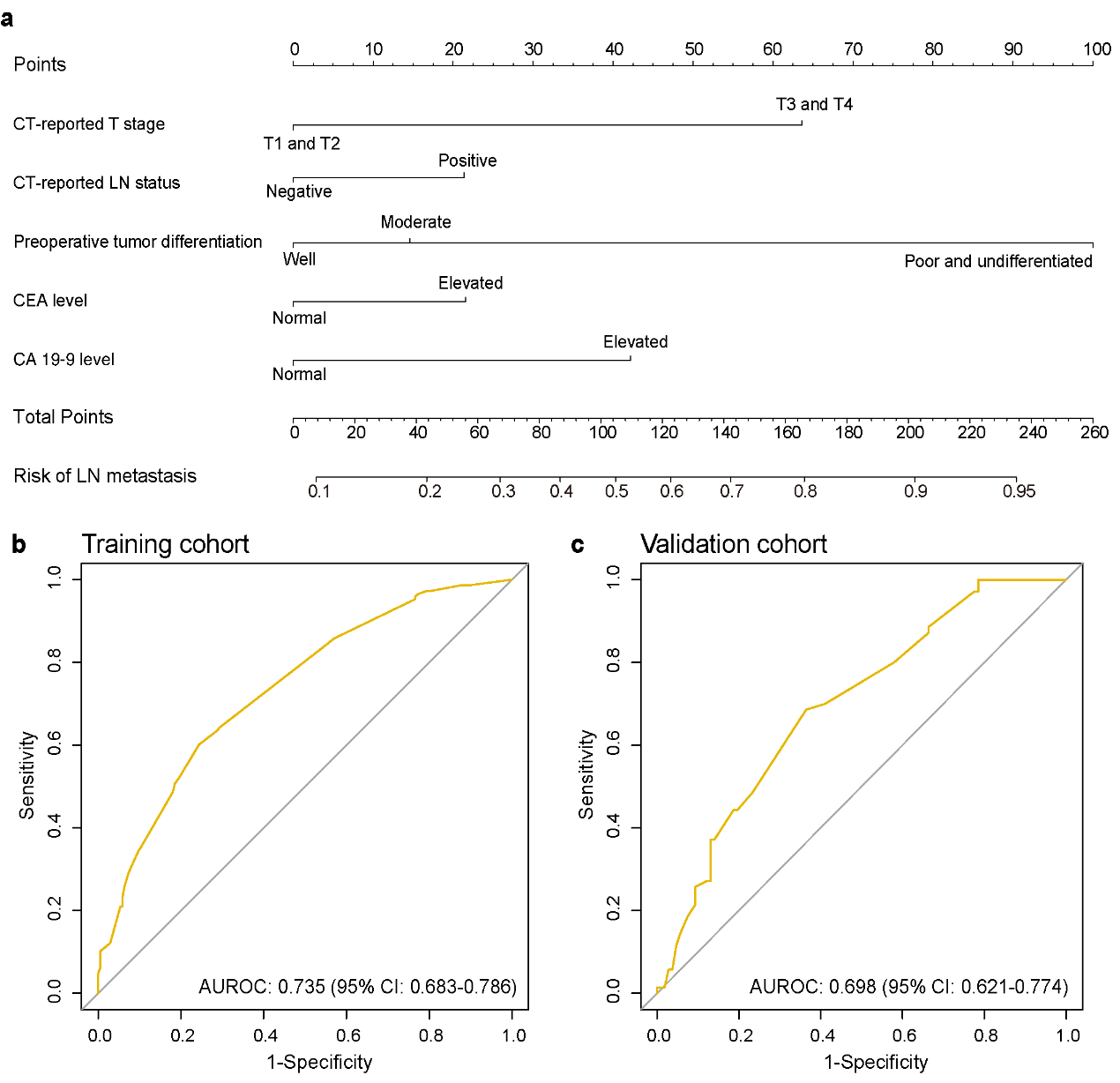
**

**Fig. S10.** Clinicopathological nomogram and its performance. **(a)** Developed clinicopathologic nomogram. **(b)** ROC curve of the clinicopathological nomogram in the training cohort. **(c)** ROC curve of the clinicopathological nomogram in the validation cohort.

**
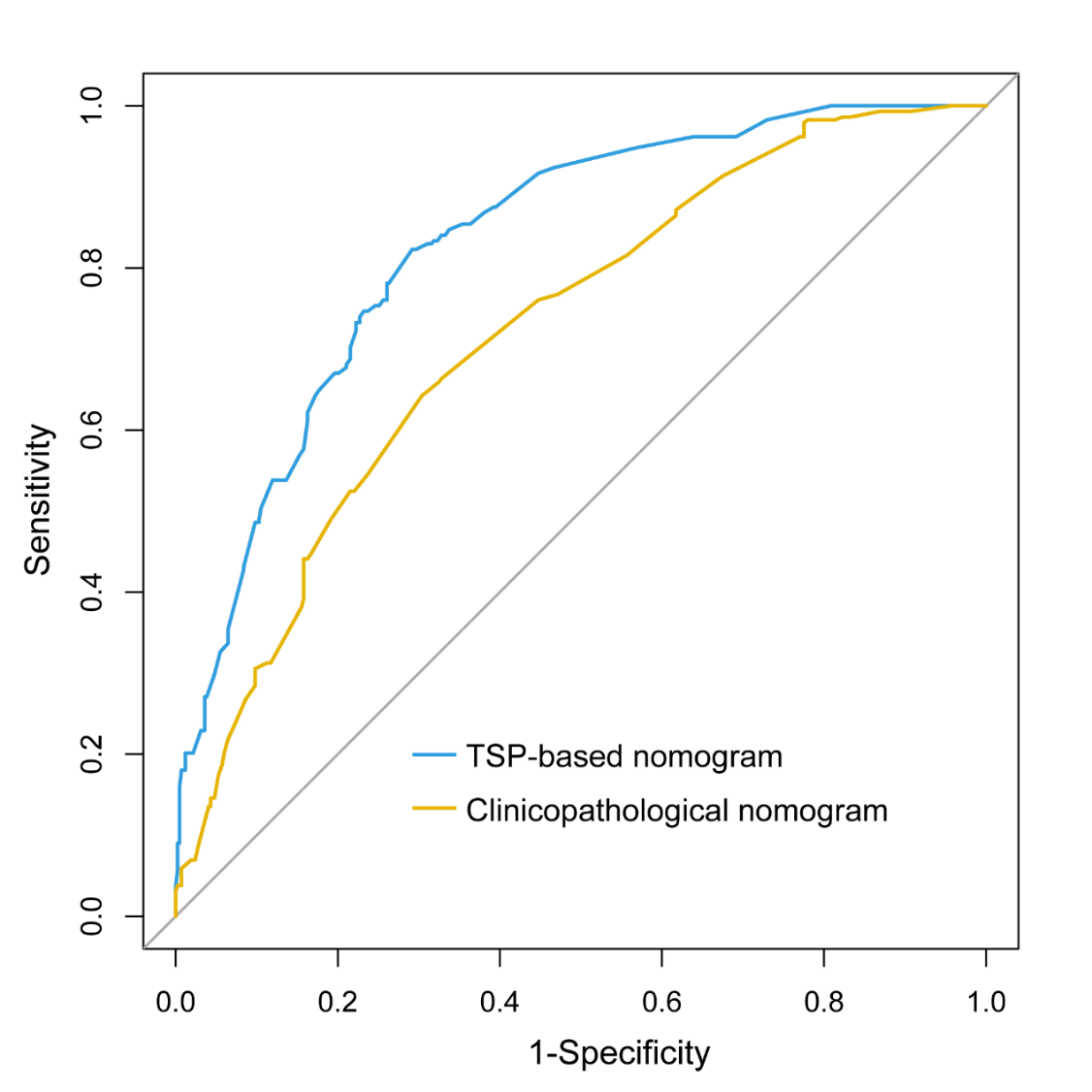
**

**Fig. S11.** Performance comparison between TSP-based nomogram and clinicopathological nomogram in all patients. The blue line represents the TSP-based nomogram, which consists of CT-reported T stage, CT-reported LN status, preoperative tumor differentiation, CEA level, CA 19-9 level and TSP, with an AUROC of 0.830 (95% CI: 0.800−0.859). The yellow line represents the clinicopathological nomogram, which consists of CT-reported T stage, CT-reported LN status, preoperative tumor differentiation, CEA level and CA 19-9 level, with an AUROC of 0.721 (95% CI: 0.684−0.758).

**Supplementary Tables**

**Table S1.** Univariate and multivariate Cox regression analyses of the preoperative predictors for OS and DFS

| **Variable** | **Univariate analysis** | ***P*** | **Multivariate analysis** | ***P*** |
| --- | --- | --- | --- | --- |
|  | HR (95% CI) |  | HR (95% CI) |  |
| ***Overall survival*** | | | | |
| **Age** | 1.002 (0.984−1.021) | 0.792 | − | − |
| **Sex** (female vs. male) | 1.211 (0.769−1.908) | 0.409 | − | − |
| **Location** (colon vs. rectum) | 1.319 (0.812−2.143) | 0.263 | − | − |
| **Preoperative histological type** (mucinous vs. adenocarcinoma) | 3.009 (1.096−8.259) | 0.033 | 1.142 (0.369−3.535) | 0.817 |
| **Preoperative tumor differentiation** |  | <0.001 |  | 0.007 |
| Well | Reference | >0.99 | Reference | >0.99 |
| Moderate | 1.145 (0.691−1.898) | 0.599 | 1.048 (0.631−1.740) | 0.855 |
| Poor and undifferentiated | 4.001 (2.112−7.579) | <0.001 | 3.018 (1.487−6.122) | 0.002 |
| **CT-reported tumor size** (>4 cm vs. ≤4 cm) | 1.322 (0.839−2.081) | 0.229 | − | − |
| **CT-reported T stage** (T3 and T4 vs. T1 and T2) | 2.428 (1.053−5.597) | 0.037 | 1.885 (0.809−4.390) | 0.142 |
| **CT-reported LN status** (positive vs. negative) | 1.549 (0.965−2.485) | 0.070 | − | − |
| **CEA level** (elevated vs. normal) | 1.751 (1.108−2.769) | 0.016 | 1.471 (0.901−2.402) | 0.123 |
| **CA 19-9 level** (elevated vs. normal) | 2.179 (1.316−3.607) | 0.002 | 1.716 (1.002−2.941) | 0.049 |
| **TSP (**high vs. low**)** | **4.289 (2.624−7.008)** | **<0.001** | **3.977 (2.428−6.514)** | **<0.001** |
| ***Disease-free survival*** | | | | |
| **Age** | 1.001 (0.986−1.015) | 0.927 | − | − |
| **Sex** (female vs. male) | 1.207 (0.851−1.712) | 0.292 | − | − |
| **Location** (colon vs. rectum) | 1.432 (0.981−2.089) | 0.063 | − | − |
| **Preoperative histological type** (mucinous vs. adenocarcinoma) | 2.024 (0.748−5.482) | 0.165 | − | − |
| **Preoperative tumor differentiation** |  | 0.008 |  | 0.059 |
| Well | Reference | >0.99 | Reference | >0.99 |
| Moderate | 1.064 (0.723−1.565) | 0.753 | 0.958 (0.649−1.413) | 0.828 |
| Poor and undifferentiated | 2.447 (1.379−4.344) | 0.002 | 1.957 (1.091−3.512) | 0.024 |
| **CT-reported tumor size** (>4 cm vs. ≤4 cm) | 1.342 (0.945−1.905) | 0.100 | − | − |
| **CT-reported T stage** (T3 and T4 vs. T1 and T2) | 2.749 (1.396−5.417) | 0.003 | 1.973 (0.986−3.947) | 0.055 |
| **CT-reported LN status** (positive vs. negative) | 1.476 (1.024−2.126) | 0.037 | 1.305 (0.893−1.908) | 0.169 |
| **CEA level** (elevated vs. normal) | 1.682 (1.181−2.397) | 0.004 | 1.416 (0.971−2.065) | 0.071 |
| **CA 19-9 level** (elevated vs. normal) | 1.597 (1.049−2.431) | 0.029 | 1.266 (0.812−1.975) | 0.298 |
| **TSP (**high vs. low**)** | **4.126 (2.841−5.993)** | **<0.001** | **3.839 (2.639−5.584)** | **<0.001** |

**Table S2.** Multicollinearity assessment of the TSP-based prediction model

| **Variable** | **Collinearity statistics** | |
| --- | --- | --- |
|  | Tolerance | Variance inflation factor |
| CT-reported T stage | 0.939 | 1.065 |
| CT-reported LN status | 0.945 | 1.058 |
| Preoperative tumor differentiation | 0.959 | 1.043 |
| CEA level | 0.853 | 1.173 |
| CA 19-9 level | 0.892 | 1.122 |
| TSP | 0.969 | 1.032 |

**Table S3.** Model performance in estimating the risk of LN status in different status

| **Variable** | **Value (95% CI)** | | |
| --- | --- | --- | --- |
|  | Training cohort | Validation cohort | Total cohort |
| Cutoff risk probability | 0.310 | 0.310 | 0.310 |
| **All stage** | | | |
| Sensitivity, % | 84.5 (71.0−93.9) | 80.0 (70.0−88.6) | 83.0 (77.8−86.5) |
| Specificity, % | 69.8 (59.5−85.4) | 72.0 (64.5−79.4) | 70.5 (66.5−75.4) |
| Accuracy, % | 75.9 (72.2− 81.6) | 75.1 (68.9−81.4) | 75.7 (72.5−78.6) |
| Negative predictive value, % | 86.1 (79.4−93.9) | 84.6 (78.4−90.8) | 85.6 (82.2−88.6) |
| Positive predictive value, % | 66.8 (61.7−78.5) | 65.1 (58.3−72.6) | 66.3 (62.7−69.9) |
| **T1 stage** | | | |
| Sensitivity, % | 50.0 (25.0−100.0) | 50.0 (50.0−100.0) | 50.0 (16.7−83.3) |
| Specificity, % | 100.0 | 100.0 | 100.0 |
| Accuracy, % | 88.2 (82.4−100.0) | 85.7 (85.7−100.0) | 87.5 (79.2−95.8) |
| Negative predictive value, % | 86.7 (81.2−100.0) | 83.3 (83.3−100.0) | 85.7 (78.3−94.7) |
| Positive predictive value, % | 100.0 | 100.0 | 100.0 |
| **T2 stage** | | | |
| Sensitivity, % | 77.8 (55.6−94.4) | 50.0 (16.7−83.3) | 70.8 (50.0−87.5) |
| Specificity, % | 80.4 (67.4−91.3) | 95.0 (85.0−100.0) | 84.9 (75.8−92.4) |
| Accuracy, % | 79.7 (68.8−89.1) | 84.6 (73.1−96.2) | 81.1 (72.2−88.9) |
| Negative predictive value, % | 90.5 (82.2−97.5) | 86.4 (78.3−95.2) | 88.9 (82.6-95.2) |
| Positive predictive value, % | 61.1 (46.7−77.8) | 75.0 (33.3−100.0) | 63.0 (48.5-79.0) |
| **T3 stage** | | | |
| Sensitivity, % | 76.9 (61.5−92.3) | 86.7 (66.7−100.0) | 80.5 (68.3−92.7) |
| Specificity, % | 70.2 (57.5−83.0) | 71.4 (53.6−85.7) | 70.7 (60.0−81.3) |
| Accuracy, % | 72.6 (61.6−82.2) | 76.7 (65.1−88.4) | 74.1 (65.5−81.9) |
| Negative predictive value, % | 84.7 (75.0−94.1) | 91.3 (80.0−100.0) | 86.9 (79.9−94.6) |
| Positive predictive value, % | 59.0 (47.6−71.4) | 61.9 (50.0−77.8) | 60.0 (51.0−70.6) |
| **T4 stage** | | | |
| Sensitivity, % | 89.0 (82.0−95.0) | 83.0 (72.3−93.6) | 87.1 (81.6−92.5) |
| Specificity, % | 60.6 (51.5−70.7) | 61.1 (48.2−74.1) | 60.8 (52.9−68.0) |
| Accuracy, % | 74.9 (68.8−80.4) | 71.3 (62.4−79.2) | 73.7 (69.0−78.3) |
| Negative predictive value, % | 84.7 (76.6−92.2) | 80.9 (70.0−90.9) | 83.2 (77.0−89.2) |
| Positive predictive value, % | 69.6 (64.1−75.4) | 65.0 (57.4−73.6) | 68.1 (63.9−72.4) |
| **cN0 subgroup** | | | |
| Sensitivity, % | 71.4 (57.1−83.3) | 79.2 (62.5−91.7) | 74.2 (63.6−83.3) |
| Specificity, % | 83.5 (75.8−90.1) | 73.8 (62.3−83.6) | 79.6 (73.0−85.5) |
| Accuracy, % | 79.7 (72.9−85.7) | 75.3 (65.9−84.7) | 78.0 (72.0−83.0) |
| Negative predictive value, % | 86.4 (80.9−91.8) | 90.0 (82.9−96.2) | 87.8 (83.1−92.0) |
| Positive predictive value, % | 66.7 (56.3−78.1) | 54.3 (43.2−67.7) | 61.0 (53.3−69.6) |

| **Variable** | **Risk subgroup** | | | | | | | | | | | | | | | | |
| --- | --- | --- | --- | --- | --- | --- | --- | --- | --- | --- | --- | --- | --- | --- | --- | --- | --- |
|  | Training cohort | | | | ***P*** | Validation cohort | | | | ***P*** | Total cohort | | | | | ***P*** | |
|  | High risk | | Low risk | |  | High risk | | Low risk | |  | High risk | Low risk | | | |  |  |
| **T stage, no. (%)** | | | | | | | | | | | | | | | | | |
| T1 | 2 (11.8) | | 15 (88.2) | | <0.001 | 1 (14.3) | | 7 (85.7) | <0.001 | | 3 (12.5) | | 21 (87.5) | | <0.001 | | |
| T2 | 23 (35.9) | | 41 (64.1) | |  | 4 (15.4) | | 22 (84.6) |  |  | 27 (30.0) | | 63 (70.0) | |  |  |  |
| T3 | 34 (46.6) | | 39 (53.4) | |  | 21 (48.8) | | 22 (51.2) |  |  | 55 (47.4) | | 61 (52.6) | |  |  |  |
| T4 | 128 (64.3) | | 71 (35.7) | |  | 60 (59.4) | | 41 (40.6) |  |  | 188 (62.7) | | 112 (37.3) | |  |  |  |
| **cN0 subgroup, no. (%)** | | | | | | | | | | | | | | | | | |
| Positive LN metastasis | 30 (71.4) | 15 (16.5) | | <0.001 | | 19 (79.2) | 16 (26.2) | | | <0.001 | 49 (74.2) | | | 31 (20.4) | | | <0.001 |
| Negative LN metastasis | 12 (28.6) | 76 (83.5) | |  |  | 5 (20.8) | 45 (73.8) | | |  | 17 (25.8) | | | 121 (79.6) | | |  |

**Table S4.** Distribution of nomogram-predicted LN metastasis risk in T stage and cN0 subgroup

| **Variable** | **Univariate analysis** | ***P*** | **Multivariate analysis** | ***P*** |
| --- | --- | --- | --- | --- |
|  | OR (95% CI) |  | OR (95% CI) |  |
| **Age** | 0.995 (0.798−1.013) | 0.601 | − | − |
| **Sex** (female vs. male) | 1.438 (0.931−2.220) | 0.101 | − | − |
| **Location** (colon vs. rectum) | 1.150 (0.745−1.777) | 0.528 | − | − |
| **Preoperative histological type** (mucinous vs. adenocarcinoma) | 3.549 (0.679−18.548) | 0.133 | − | − |
| **Preoperative tumor differentiation** |  | 0.002 |  | 0.010 |
| Well | Reference | >0.99 | Reference | >0.99 |
| Moderate | 1.551 (0.919−2.616) | 0.100 | 1.391 (0.795−2.436) | 0.248 |
| Poor and undifferentiated | 12.628 (2.828−56.391) | 0.001 | 9.574 (2.053−44.646) | 0.004 |
| **CT-reported tumor size** (>4 cm vs. ≤4 cm) | 1.037 (0.680−1.583) | 0.865 | − | − |
| **CT-reported T stage** (T3 and T4 vs. T1 and T2) | 4.982 (2.363−10.502) | <0.001 | 4.206 (1.873−9.449) | 0.001 |
| **CT-reported LN status** (positive vs. negative) | 2.015 (1.283−3.163) | 0.002 | 1.620 (0.987−2.659) | 0.056 |
| **CEA level** (elevated vs. normal) | 2.706 (1.709−4.284) | <0.001 | 1.627 (0.974−2.716) | 0.063 |
| **CA 19-9 level** (elevated vs. normal) | 3.751 (2.072−6.792) | <0.001 | 2.592 (1.342−5.004) | 0.005 |

**Table S5.** Univariate and multivariate logistic analyses in the training cohort without TSP
